# The stage of seed development influences iron bioavailability in pea (*Pisum sativum* L.)

**DOI:** 10.1038/s41598-018-25130-3

**Published:** 2018-05-02

**Authors:** Katie L. Moore, Ildefonso Rodríguez-Ramiro, Eleanor R. Jones, Emily J. Jones, Jorge Rodríguez-Celma, Kirstie Halsey, Claire Domoney, Peter R. Shewry, Susan Fairweather-Tait, Janneke Balk

**Affiliations:** 1 School of Materials, University of Manchester, Manchester, M13 9PL UK; 2 Norwich Medical School, University of East Anglia, Norwich, NR4 7UQ UK; 3 Department of Biological Chemistry, John Innes Centre, Norwich, NR4 7UH UK; 4 School of Biological Sciences, University of East Anglia, Norwich, NR4 7TJ UK; 5 Department of Plant Sciences, Rothamsted Research, Harpenden, AL5 2JQ UK; 6 Department of Metabolic Biology, John Innes Centre, Norwich, NR4 7UH UK

## Abstract

Pea seeds are widely consumed in their immature form, known as garden peas and petit pois, mostly after preservation by freezing or canning. Mature dry peas are rich in iron in the form of ferritin, but little is known about the content, form or bioavailability of iron in immature stages of seed development. Using specific antibodies and in-gel iron staining, we show that ferritin loaded with iron accumulated gradually during seed development. Immunolocalization and high-resolution secondary ion mass spectrometry (NanoSIMS) revealed that iron-loaded ferritin was located at the surface of starch-containing plastids. Standard cooking procedures destabilized monomeric ferritin and the iron-loaded form. Iron uptake studies using Caco-2 cells showed that the iron in microwaved immature peas was more bioavailable than in boiled mature peas, despite similar levels of soluble iron in the digestates. By manipulating the levels of phytic acid in the digestates we demonstrate that phytic acid is the main inhibitor of iron uptake from mature peas *in vitro*. Taken together, our data show that immature peas and mature dry peas contain similar levels of ferritin-iron, which is destabilized during cooking. However, iron from immature peas is more bioavailable because of lower phytic acid levels compared to mature peas.

## Introduction

Peas, beans, soybeans and other pulses are seed crops that belong to the legume family. They are naturally high in iron and may provide an important source of iron for human nutrition when consumed as a staple or vegetable. The absorption of iron from plant foods varies but is generally low (0–15%) compared to meat (30%) due to a number of factors^1^. While meat is rich in haem iron, plants contain mostly non-haem iron, a heterogenous mixture of iron species bound to organic molecules or proteins^2^. Non-haem iron is generally released during digestion and will bind to dietary chelators that act as inhibitors of absorption, such as phytic acid (myo-inositol hexakisphosphate) and polyphenols^1^.

A unique property of legume seeds is that a large proportion of the iron is stored in the form of ferritin, forming a 24-mer protein shell that can store up to 4500 iron atoms^3–5^. Homology modelling and crystal structures of soybean ferritin showed that plant ferritin has high structural similarity to vertebrate ferritin, except that the protein shell of plant ferritins is formed of identical protein chains rather than H- and L-chains^6^. The ferritin particles are ~12 nm in diameter with a cavity of 8 nm. Ferrous iron is oxidised by an intrinsic ferroxidase activity and stored as ferrihydrite [(Fe^3+^)_2_O_3_•nH_2_O]^7^. In vegetative tissues such as leaves, ferritin plays an important biological role in capturing excess iron to protect plant cells from reactive oxygen species generated by Fe^2+^/Fe^3+^ cycling^8^. In seeds, ferritin serves as an iron store for the seedling upon germination, in addition to iron stored in vacuoles^2^. The relative amount of iron stored in ferritin versus vacuoles in the seeds depends on the plant species, ranging from 2% ferritin-iron in Arabidopsis^8^ to 15–69% ferritin-iron in legumes such as common bean, peas and lentils^4^. Plant ferritins are targeted to the plastids, with minor amounts localized in the mitochondria^5^. This means that ferritin-iron is surrounded by two intracellular membranes as well as a plasma membrane and the plant cell wall, which may affect the release of iron from ferritin during digestion.

Legumes are well characterized with respect to changes in starch, protein and lipids during seed maturation^9^, however little is known about the translocation of iron into developing seeds^10^. A recent study of pea (*Pisum sativum*) showed that the mother plant transports ferric-malate/citrate complexes via the seed coat to the embryo, which in turn secretes ascorbate to reduce ferric iron to its ferrous form for uptake^11^. The fate of iron after uptake into the developing embryo, including how and when it is mineralized into ferritin, is not known.

As a food crop, peas are mostly grown for dry seeds, but also for ‘green’ (immature) peas, with Asia, the Americas and Europe being the main producers (www. fao.org.faostat). The UK is the largest producer of pea seeds for freezing in Europe, with a relatively static area devoted to the production of this crop at ~35,000 ha, yielding up to 5 t/ha or upwards of 150 M kg of frozen product (www.pgro.org; ukpeaandbeans.co.uk). The area cultivated for immature pea seeds is limited by proximity to processing factories and the associated contracts to meet a defined market. In contrast, the area in the UK devoted to the production of mature dry peas is more variable, ranging from ~35,000 to almost 50,000 ha, with yields of up to 5 t/ha. The season for the immature pea seed crop is short (~10 weeks), whereas mature dry seeds can be stored and then processed the whole year round. With the development of modern food preservation techniques, namely canning in the 19^th^ century and freezing in the 20^th^ century, the consumption of immature peas is no longer seasonal. The breeding of cultivars of pea for the immature seed industry has developed since the 19^th^ century, leading to the distinction of two classes, known as petit pois and garden peas, which differ primarily in their seed size.

With increasing interest in the provision of adequate mineral nutrients in human diets, the growing pressure to eat less meat and the prevalence of iron-deficiency anaemia, it is important to understand how crop plants accumulate iron and whether this is bioavailable. The iron in purified soybean ferritin is highly bioavailable^12^, and it has been reported that ferritin-iron remains intact during gastrointestinal digestion^13^ and may be taken up by phagocytosis^14^. However, other reports demonstrated that ferritin is completely destroyed during cooking and digestion^15^, and the released iron is taken up into mucosal cells by DMT1 (Divalent Metal Transporter 1).

In this study, we observed that the levels of ferritin-iron in immature pea seeds are similar to those of mature seeds, with clusters of ferritin-iron being located on the outside of starch grains. Ferritin protein is destabilized during cooking, including brief microwave heating of frozen peas. However, the phytic acid content is significantly lower in immature peas, resulting in the iron being more bioavailable in cooked immature peas than in mature peas.

## Results

### Accumulation of ferritin-iron during seed development

**Figure 1:**
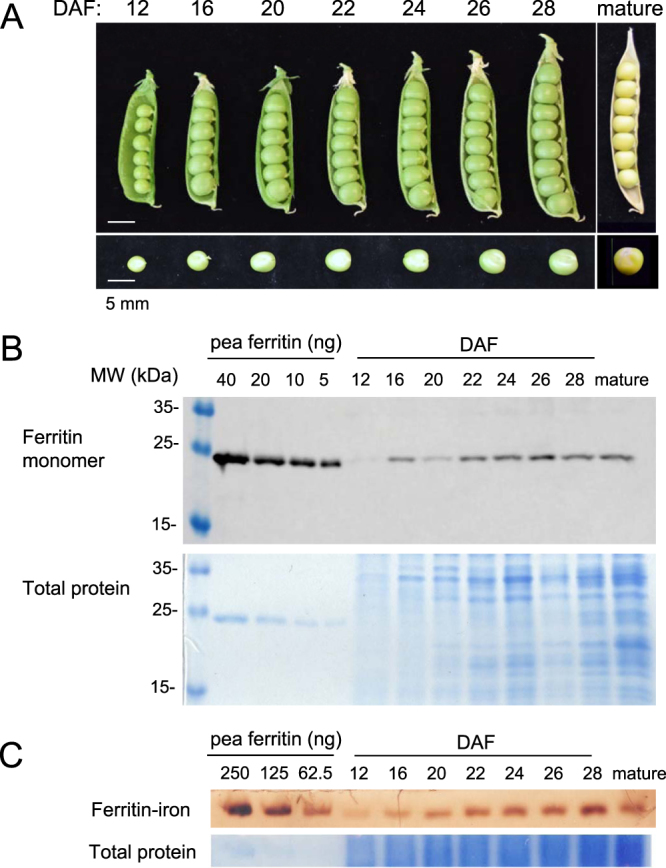
Ferritin protein and iron loading during development of pea seeds. (**A**) Images of opened seed pods at the indicated days after flowering (DAF). (**B**) Protein blot analysis of ferritin in extracts from pea seeds. A dilution series of purified pea ferritin (40 − 5 ng) was run in the four left lanes for comparison with pea extracts (5 µg protein per lane). (**C**) Iron staining associated with assembled ferritin shells. A dilution series of freshly purified pea ferritin (250–62.5 ng protein, or 55 – 14 ng Fe) and protein extracts from developing peas (20 µg protein per lane) were separated by native gel electrophoresis and stained for iron with Perls’ staining and diaminobenzidine enhancement. The data shown are representative of 4 independent time courses. A second data set is shown in Figure S2.

To investigate the accumulation of iron and ferritin during development, pea seeds were harvested every 2–4 days from 12 days after flowering (DAF) until they had matured on the vine to the dry form (Table 1, Fig. 1A). The industry standard for the harvest of immature garden peas for freezing is a tenderometer (TR) value of 100. While this measurement is an indicator of the stages of seed development in the field, it is not useful in a laboratory context. To enable comparisons with industry materials, a TR100 value was shown to correspond to seeds having ~45% (w/w) dry matter of mature seed, based on analysis of several cultivars (Figure S1). Thus, we found that under our growth conditions pea seeds harvested at 22 to 24 DAF are equivalent to garden peas provided by industry.

**Table 1:** Dry weight, protein and iron over the course of development in pea seeds.

DAF^a^	Dry weight (mg)^b^	% Dry weight of mature	Percentage protein^c^	Total Fe (µg/gDW) ± SD
12	38	11	3.9	38.3 ± 2.6
16	65	19	5.0	26.7 ± 1.3
20	125	35	5.9	30.6 ± 0.5
22^d^	158	45	4.5	27.9 ± 0.6
24^d^	160	46	4.9	30.3 ± 0.6
26	204	58	6.2	29.3 ± 0.8
28	250	71	7.2	30.0 ± 1.0
mature	351	100	13	34.2 ± 1.2

A typical developmental time course is shown here, a second data set is presented in Figure S2.

^a^Days after flowering.

^b^Average of 3 individual peas which were weighed together. The variance is 10–20%, see Figure S2.

^c^mg protein (relative to bovine serum albumin)/mg dry weight *100%.

^d^These two developmental stages have ~45% dry weight matter of mature peas, corresponding to a tenderometer value of 100, at which stage the seeds are harvested for frozen garden peas.

The iron concentration measured by ICP-OES was constant on a dry weight basis during seed growth, at approximately 30 µg/g (Table 1). However, as the seeds increased in size and dry weight, the iron content per seed increased by more than 7-fold, from 1.7 ± 0.08 µg iron per seed at 16 DAF to 12 ± 0.4 µg iron in individual mature seeds. Accumulation of ferritin protein was investigated by protein blot analysis using antibodies raised against pea ferritin (Figure S3). At 12 DAF ferritin protein was barely detectable, but increased ~10-fold until maturity (Fig. 1B; Figure S2). To determine the amount of iron loaded into ferritin protein shells, the same protein extracts were separated by native polyacrylamide gel electrophoresis and stained for iron with Perls’ staining enhanced with diaminobenzidine (Fig. 1C). The amount of iron associated with assembled ferritin shells (~550 kDa) increased over time, and correlated with the increase in ferritin monomer (Figure S4).

### Ferritin-iron is localized to the periphery of starch-filled plastids

**Figure 2:**
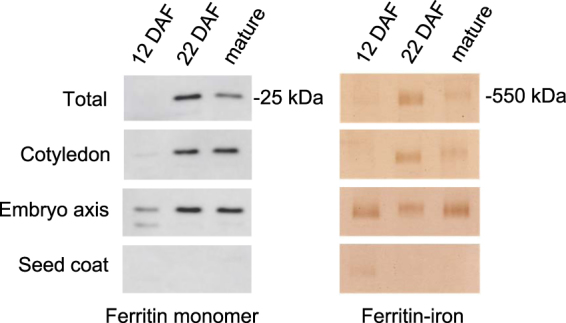
Ferritin levels in cotyledons, embryo axis and seed coat. Protein extracts (20 µg) from total pea seeds (JI1194), cotyledons, embryo axis and seed coat at three stages of development were separated by SDS-PAGE for immunoblot analysis (left panels) or by native PAGE for iron staining (right panels). The results are representative of 3 independent biological replicates, and each sample was prepared from 2–3 seeds or tissues.

To investigate where ferritin accumulates in developing seeds, the embryo (comprising of two cotyledons and the embryo axis) and seed coat were hand dissected. Protein extracts were analysed by protein blot analysis with antibodies against pea ferritin and also by in-gel iron staining. Ferritin loaded with iron was highest on a total protein basis at 22 DAF and abundant in both parts of the embryo. By contrast, only low amounts of ferritin were found in the seed coat of young seeds (12 DAF) and it was undetectable at later stages of seed development (Fig. 2). This is in agreement with findings reported for the pea cultivar Sparkle^16^.

To obtain information on the subcellular localization of iron in peas, high-resolution secondary ion mass spectrometry (NanoSIMS) images were obtained from peas at 22–24 DAF. This technique uses an ion beam to bombard the surface of a material, in this case thin sections of resin-embedded plant material, resulting in sputtering of the top few atomic layers and ejection of ions which are analysed in a mass spectrometer^17^. Tissue samples from the cotyledons, embryo axis and seed coat were high pressure frozen, fixed, embedded in resin and sectioned. For each anatomical part two areas were imaged to show different cell types. The very recognizable parenchyma cells accumulate protein, lipid and starch in vesicles or storage organelles and are particularly large (>0.1 mm) in cotyledons. Iron was mapped by NanoSIMS as ^56^Fe^16^O^−^ rather than ^56^Fe^+^, as imaging negative ions with the Cs^+^ ion source gives higher spatial resolution and allows simultaneous mapping of ions such as ^12^C^14^N^−^ and ^31^P^16^O^−^ (ref.^18^). The NanoSIMS is not able to give information regarding the speciation or bonding of the iron directly.

**Figure 3:**
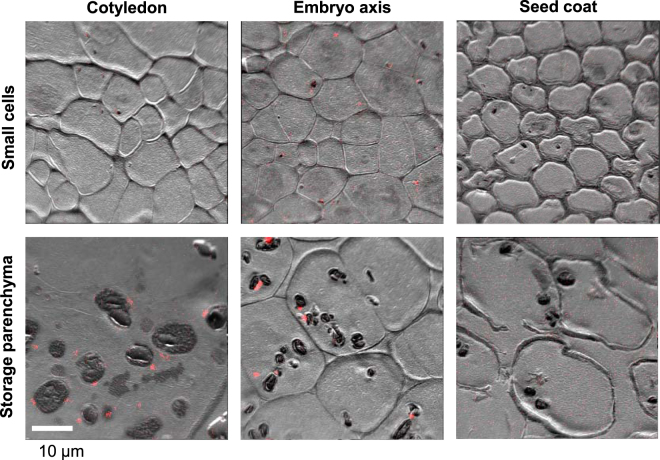
Discrete spots of iron are associated with amyloplasts in pea seeds. NanoSIMS localization of iron in sections of immature pea seeds (JI1194) at 22–24 DAF. The images are representative of 6 analysed regions from the cotyledon, 6 from the embryo axis and 2 regions from the seed coat, showing either small cell types or large storage parenchyma cells. The ion-induced secondary electron image is shown in grey scale and the ^56^Fe^16^O^−^ signal is shown superimposed in red. Images with the ^56^Fe^16^O^−^ signal only are shown in Figure S5. The dark oval features observed in the larger cells are starch-filled amyloplasts.

NanoSIMS analysis showed that iron was present as dense spots on the outside of starch-filled amyloplasts in the cotyledons and embryo axis. This can be seen in the colour merge images in Fig. 3 where iron is shown in red and the ion-induced secondary electron (SE) image is presented in grey scale, clearly showing the shape and structure of the cells. The dark grey oval shapes are the amyloplasts. No dense iron spots were found in the seed coat, only a diffuse localization which is more apparent without the superimposed SE image (Figure S5). Integration of the ^56^Fe^16^O^−^ counts gave 0.016 counts/pixel (n = 2 mapped regions) which is similar to 0.024 counts/pixel in cotyledons (n = 6 mapped regions). The latter finding is supported by a study reporting a total iron concentration of ~60 µg/gDW but no ferritin protein in the seed coat of developing peas^16^. The embryo axis contained an average of 0.098 ^56^Fe^16^O^−^ counts/pixel (n = 6 mapped regions).

**Figure 4:**
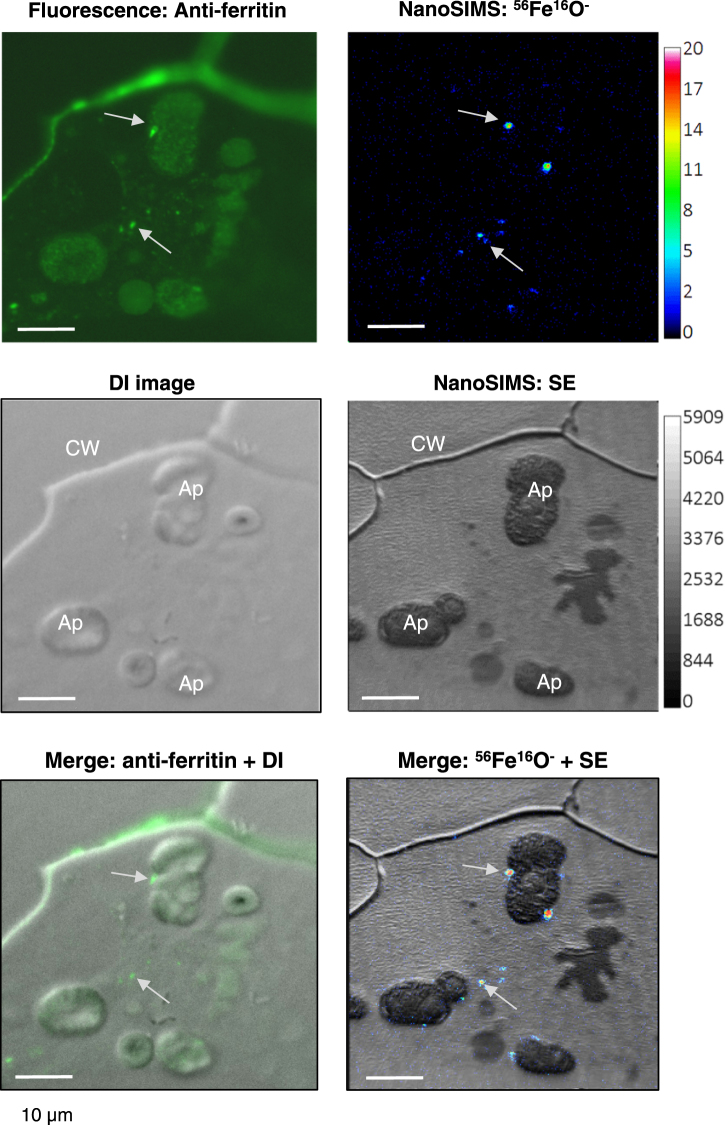
Corresponding localization of iron and ferritin protein. Adjacent thin sections of cotyledons from immature pea seeds (JI1194) at 22–24 DAF were labelled with antibodies against pea ferritin followed by Alexa-Fluor conjugated secondary antibodies (top left, green) or imaged for ^56^Fe^16^O^−^ ions using NanoSIMS (top right, with colour scale for the intensity of the signal). The differential interference (DI) image of the immunolabelled section and the secondary electron (SE) image for NanoSIMS show the cell walls and organelles. CW, cell wall; Ap, amyloplast. The images are representative of 2 different regions for which 2 cells each were imaged.

The high density of the iron in spots associated with the amyloplasts in cotyledons and embryo axis suggests that these spots represent mineralized iron cores of ferritin. To confirm the presence of ferritin protein, a section from the cotyledon immediately adjacent to that analysed by NanoSIMS was immunolabelled with antibodies against pea ferritin. Positive fluorescence staining was found in the cell wall and in dense spots (Fig. 4). One dense spot on the outside of a starch grain (top arrow) and several spots clustered together towards the middle of the cell (bottom arrow) clearly corresponded in location in the NanoSIMS and the immunolabelled sections. For the cluster of spots, a starch grain is likely present outside the focal plane. Strong immunolabelling of the cell wall was also observed, probably due to binding of the antibody to epitopes unrelated to ferritin (Figure S3). Based on the observed distribution of the spots and the corresponding localization between the two techniques, we conclude that the dense iron spots are indeed iron-loaded ferritin. Furthermore, the absence of these spots in the seed coat correlates with the absence of ferritin shown by protein blot analysis (Fig. 2). It is important to note that the spots measure approximately 0.5–1 µm, which is many orders of magnitude larger than the 8-nm iron core of ferritin and the 100–150 nm NanoSIMS beam size. Therefore, the spots are likely to represent clusters of ferritin-iron.

### Ferritin is not colocalized with phytic acid

**Figure 5:**
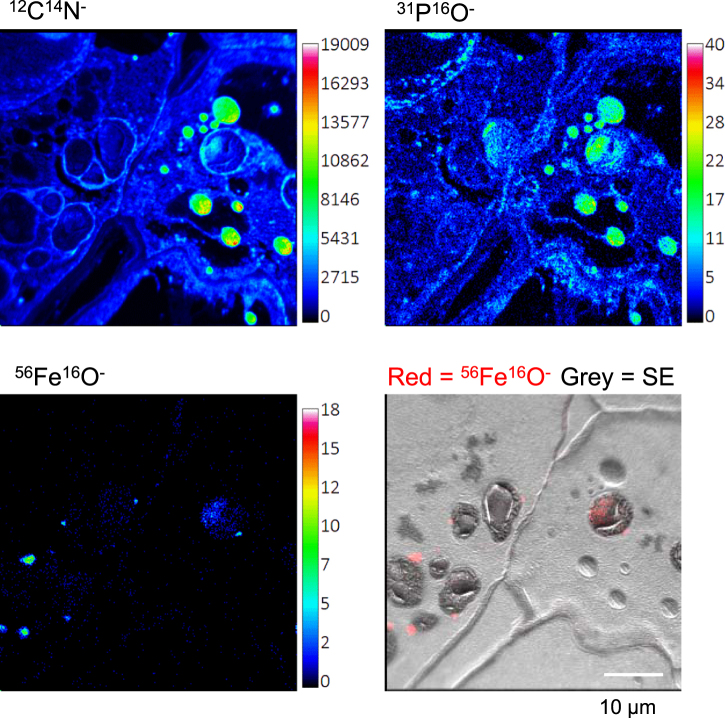
Colocalization of Fe with protein and PO compounds. NanoSIMS images of ^12^C^14^N (top left), ^31^P^16^O^−^ (top right), and ^56^Fe^16^O^−^ (bottom two images) in cotyledons of immature pea seeds (JI1194) at 22–24 DAF. The images show part of neighbouring cells representative of the cotyledons. The image bottom left shows the dense iron spots on the outside of amyloplasts as well as the diffuse pattern of iron inside the amyloplasts (overlay of ^56^Fe^16^O^−^ in red and secondary electrons, SE, in grey scale). The images are representative of 4 different regions each showing colocalization of Fe with PO compounds.

To obtain information about molecules that co-localize with iron, ^12^C^14^N^−^ and ^31^P^16^O^−^ were mapped by NanoSIMS simultaneously with ^56^Fe^16^O^−^. Analysis of ^12^C^14^N^−^ serves as a proxy for amino acids and protein and shows the morphology of the cells, while ^31^P^16^O^−^ can be used as proxy for phosphate, phytic acid and phospholipids^18^. Pea seeds are high in protein that is stored in small vesicles or protein bodies, and these can be seen on the right-hand side of the cell as circular features in the ^12^C^14^N^−^ image (Fig. 5). ^12^C^14^N^−^ also marks protein-rich membranes, such as those surrounding the amyloplasts. ^31^P^16^O^−^ co-localized with ^12^C^14^N^−^ in small vesicles on the right of the cell, but is also found within the amyloplasts, together with a diffuse pattern of ^56^Fe^16^O^−^. Thus, it is possible that some iron, but not ferritin-iron, is in direct contact with phosphate or phytic acid in immature pea seeds.

### Ferritin protein in peas is destabilized by cooking

**Figure 6:**
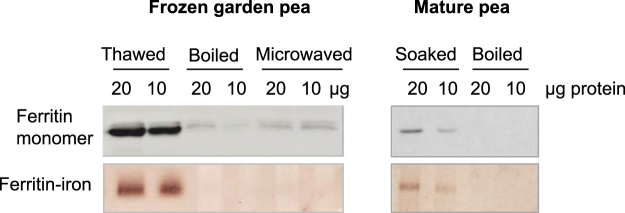
Ferritin in immature frozen peas and mature dry pea seeds is destabilized by different cooking methods. Frozen garden peas were boiled or microwaved, and mature dry peas were soaked and boiled. Protein extracts were analysed for ferritin monomer by protein blot analysis (top panel) and for iron associated with ferritin by in-gel Perls’/DAB staining (lower panel). The data are representative of 2 biological repeats.

Purified ferritin protein from mature pea and soybean seeds is denatured by heat treatment during boiling for 50 min, the time it takes to cook dry legume seeds until soft^15^. By contrast, garden peas are sold mostly in frozen form and the cooking instructions on packs suggest microwaving or simmering the peas from frozen for 2–4 min. We tested the effect of brief microwave heating on ferritin, and compared this to the longer cooking period of dry mature peas. After cooking, the peas were cooled on ice and proteins were extracted for analysis of ferritin protein and iron associated with ferritin. Protein blot analysis of the ferritin monomer showed that little ferritin protein was detectable after cooking (Fig. 6), presumably because the protein was degraded or insoluble in the extraction buffer. In-gel iron staining confirmed the absence of a higher molecular weight assembly of ferritin with iron. Thus, even a short heat treatment results in the release of iron from ferritin in pea seeds.

### Iron is more bioavailable in cooked garden peas than in mature peas

**Figure 7:**
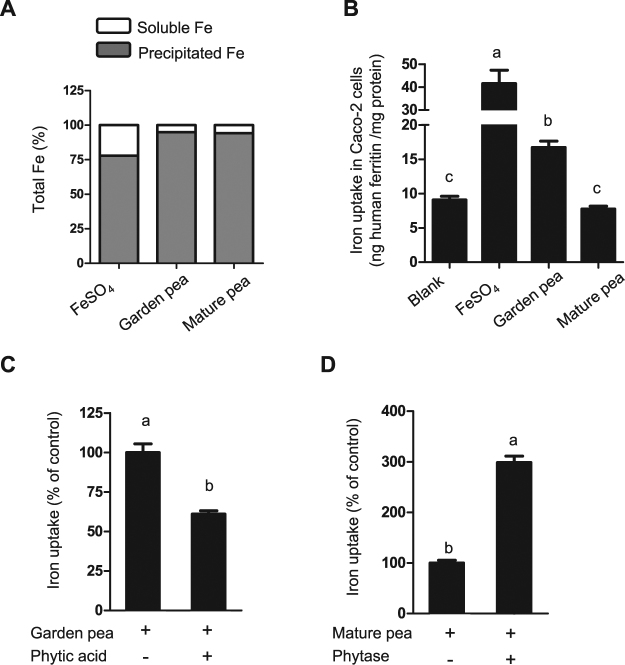
Iron in cooked immature garden peas is more bioavailable than in mature peas. (**A**) Percentage of soluble and precipitated iron in the final step of the simulated gastrointestinal digestion. (**B**) Iron uptake in Caco-2 cells exposed to the digestates of microwaved (immature) garden peas and boiled mature peas. (**C**) Percentage of change in iron uptake into Caco-2 cells after adding phytic acid to the simulated digestion of garden peas, and (**D**) after adding phytase enzyme to the simulated digestion of mature peas. Data represent means ± SEM from 3–4 different simulated digestions which were each applied in triplicate to Caco-2 cells. Means without a common letter differ (*p* < 0.05, using one-way ANOVA for (B) and Student *t*-test in (**C** and **D**).

To investigate whether the developmental stage of pea seeds affects the supply of available iron for human nutrition, the uptake of iron from microwaved garden peas and boiled mature peas was determined using Caco-2 cells, a widely used cellular model of the small intestine^19^. First, the cooked peas were subjected to *in vitro* gastrointestinal digestion, simulating the gastric conditions at pH 2.0 in the presence of pepsin, followed by intestinal conditions at pH 7 with pancreatin and bile extract. For the digestion, ascorbate was added at a 10-fold excess of total iron as determined by ICP-OES. Ascorbate is a standard component of the bioavailability assay, necessary to enable iron uptake into Caco-2 cells^19^. The digestates were centrifuged to remove insoluble debris. Iron determination showed that only 6% of the total iron was present in the soluble fraction of the digestates from both types of peas (Fig. 7A). Caco-2 cells were incubated with the soluble digestate fraction and iron uptake was assessed after 24 hours, using human ferritin content as a surrogate measure of iron uptake into cells. A blank assay with all digestion components but no iron was used to measure background levels of ferritin, and a reference dose of 50 µM FeSO_4_ was used to determine the iron uptake capacity of the cells. Interestingly, iron was absorbed from immature garden peas but not from mature peas (Fig. 7B).

Phytic acid is one of the major inhibitors of iron absorption in plant-based diets^1^ and accumulates during pea seed development^20,21^. In agreement with this developmental pattern, the phytic acid concentration was lower in cooked garden peas (7.9 mg/gDW) than in boiled mature peas (11 mg/gDW) (Table 2). This equates to molar ratios of phytic acid:iron of approximately 10 and 20 in the digestates of garden peas and cooked mature peas, respectively (Table 2). To test whether the difference in iron bioavailability is due to phytic acid, we manipulated the phytic acid content of the pea digestates (Fig. 7C,D). Addition of phytic acid to the simulated digestion of garden peas to a molar ratio of 20 significantly decreased iron uptake in Caco-2 cells by 40% (from an average of 16.7 to 10.2 ng ferritin/mg protein). Conversely, the addition of phytase enzyme to the simulated digestion of boiled mature peas significantly increased the iron uptake in Caco-2 cells by 3-fold (from an average of 7.7 to 23.1 ng ferritin/mg protein). Taken together, these results indicate that the iron in immature garden peas is more bioavailable than in mature peas, primarily because of lower phytate levels in immature garden peas.

**Table 2:** Iron and phytic acid concentrations in pea samples.

	Cooked peas^a^		Digestate		
	Fe (µg/gDW)	Phytic acid (mg/gDW)	Pea powder (g/15 ml)^b^	Phytic acid (mg/g)	Phytic acid:Fe molar ratio
Microwaved garden peas	65.9	7.92	0.63	5.01	10.17
Boiled mature peas	46	11.18	0.91	10.0	20.56

^a^Lyophilized prior to analysis of iron and phytic acid.

^b^The amount of each sample was adjusted prior to the simulated digestion to achieve a final concentration of 50 µM iron in the volume of digestion.

## Discussion

Plant foods such as cereal grains, pulses and vegetables contain significant amounts of iron, but absorption is lower than from meat. Iron bioavailability studies have mainly focused on staple crops, such as cereals, while vegetables are less well studied. Pea seeds are consumed in their immature form as a vegetable, and in dried form as a staple. In addition, peas are rich in ferritin, an iron-protein species that has received considerable attention as a nutritional iron source. The aim of this study was to determine the content, form, and bioavailability of iron in immature peas.

We observed that the total iron concentration per gram dry weight is constant during pea seed development (Table 1), but from 12 days after flowering ferritin-iron starts to accumulate (Fig. 1). Hoppler and colleagues^4^ used isotope dilution mass spectrometry to determine the percentage of iron in ferritin in a range of legume seeds and found that in two different types of locally sourced mature pea seeds ferritin-iron accounted for 52% and 62% of total iron. These values are lower than the figure of 90% often quoted in review papers, derived from in-gel iron staining using commercially available horse spleen ferritin as standard^16^. We have noticed that iron is released from purified ferritin during storage (unpublished results), therefore a ferritin standard should be prepared fresh and ideally from the same biological species. Isotope dilution mass spectrometry uses ^57^Fe as an internal standard, removing the requirement of a separate calibration standard. Therefore, the value of 50–60% of total iron stored in ferritin in pea seeds is likely to be more accurate, although there may be differences depending on pea variety and growth conditions, which has not systematically been investigated.

Using NanoSIMS analysis, we provide unprecedented detail on the subcellular distribution of iron and ferritin. The resolution of NanoSIMS is approximately 100–150 nm, whereas other mineral imaging techniques have lower resolution such as ~1 µm for Particle-Induced X-ray Emission (µPIXE)^22^, >10 µm for Perls’/DAB staining of sections^23^ and 100 µm for Synchrotron Radiation X-ray Fluorescence (SXRF)^24^. While the association of ferritin with amyloplasts has been shown in common bean^25^, the higher resolution of NanoSIMS clearly indicates a clustering of hundreds of iron-loaded ferritin shells: the iron core of ferritin is 8 nm, whereas the ferritin clusters imaged by NanoSIMS are approximately 1 µm in diameter. We used high pressure freezing for tissue fixation which gives high quality preservation and retention of small molecules (which may be lost with other fixation methods), therefore the clustering is unlikely to be an artefact of sample preparation. Why ferritin is not regularly distributed throughout or on the periphery of plastids is an interesting question which requires further research. If other proteins facilitate the clustering, then a mutagenesis approach may identify “ferritin clustering mutants” that could reveal the biological relevance, if any, of the observed distribution.

Interestingly, we did not observe accumulation of iron in the nucleolus (Fig. 3), as previously reported by Roschzttardtz and colleagues^26^ with Perls’/DAB staining, µPIXE and SXRF. Figure S6 shows additional NanoSIMS data in which the ^31^P^16^O signal marks the location of the nuclei and several nucleoli. At present we cannot explain the discrepancy, which needs further investigation such as a careful side-by-side comparison of sample preparation methods.

NanoSIMS analysis also revealed that the PO content of ferritin-iron spots is very low (Fig. 5), indicating that the ferrihydrite core is similar to that of animals, and not rich in phosphate as previously suggested^5^.

The abundance of iron in the form of ferritin, its association with starch and separation from PO-rich vesicles do not appear to influence iron bioavailability: Cooking of immature peas in a microwave oven for a few minutes or conventional boiling both denatured pea ferritin in immature peas, and is likely to result in the release of iron. The longer boiling times required for dry peas and other legume seeds also completely denatured ferritin (Fig. 6, ref.^15^). However, during the preparation of tofu from soybeans, iron-loaded ferritin was shown to be partially preserved, surviving boiling for 10 min and a further heat treatment at 80 °C^27^.

Our results indicated that the main determinant of iron bioavailability in immature and mature peas is phytic acid. The negative effect of phytic acid on iron absorption has been shown in many studies but, specifically for peas, two low-phytate lines showed a 1.5–2-fold increase in iron uptake in Caco-2 cells^20^. The lines were obtained by breeding and the phytic acid:iron ratio was reduced from 20 to 10 compared to the original cultivar, Bronco. These numbers correspond well to our studies, in which immature peas had a phytic acid:iron ratio of 10 compared with 20 in mature peas, with significantly improved iron bioavailability in the former (Fig. 7). The difference in the phytic acid:iron ratio between immature and mature peas (Table 2) indicates that the conversion of inorganic phosphate to phytate occurs relatively late in pea seed development, whereas the levels of iron are constant on a dry weight basis (Table 1). It would be interesting to determine iron bioavailability in immature stages of the low-phytate lines, as the phytic acid:iron ratio is expected to be around 5. In contrast, soaking pea seeds overnight has little effect on lowering the phytic acid content^28^. Plants do induce endogenous phytases to release phosphate from phytic acid, but this process starts after rehydration of the seeds. The inhibitory effect of phytic acid has also been shown in humans from short-term isotope absorption studies of mixed meals containing beans (*Phaseolus vulgaris*). Decreasing phytic acid appears to be the best way to increase uptake from high-iron varieties of bean^29^.

In summary, the high-resolution chemical imaging technique NanoSIMS has provided new insights into the distribution of iron and ferritin in developing pea seeds. We show that immature peas are rich in iron but have less phytic acid, correlating with higher iron bioavailability in Caco-2 cells. Immature peas are already widely consumed, but they can be further promoted as a source of bioavailable iron.

## Methods

### Reagents

Chemicals, enzymes and hormones were purchased from Sigma-Aldrich, UK unless otherwise stated.

### Plant material and growth

Pea seeds (*Pisum sativum* L.) representing a typical garden pea variety (genotype *rr*, accession number JI1194) were obtained from the John Innes Germplasm Resources Unit (www.jic.ac.uk/ germplasm/index.htm). Seeds were planted in peat-based compost plus grit, and grown in a greenhouse with partial temperature control. Plants were watered as required. Frozen garden peas were obtained from a local supermarket and dry mature pea seeds (*Pisum sativum* L. cv Sakura, marketed as marrowfat peas) were provided by Wherry & Sons (Bourne, UK).

### Quantitative analysis of iron, phosphorus and phytic acid

The concentrations of iron and phosphorus were determined using Inductively Coupled Plasma Optical Emission Spectroscopy (ICP-OES). Fresh and mature pea seeds were dried at 55 °C overnight and ground to a fine powder with a mortar and pestle. Pea flour (50 mg) was acid digested in 2 ml nitric acid (69%) and 0.5 ml hydrogen peroxide (30%) for 6 h at 95 °C. The acid digests were diluted with 25 ml MilliQ water for ICP-OES. All analyses were carried out in triplicate from separate acid digestions. For measuring the iron concentration in simulated gastrointestinal digestions of cooked peas, 0.4 ml of digest was mixed with 0.4 ml nitric acid (69%) and 0.4 ml hydrogen peroxide (30%), and incubated at room temperature for 24 h and subsequently at 40 °C overnight. Samples were diluted with 4.32 ml MilliQ water to 5% nitric acid prior to ICP-OES.

The concentrations of phytic acid and total phosphorus were determined using a commercially available kit (K-PHYT 11/15 from Megazyme, Ireland).

### In-gel iron staining

Pea ferritin was purified as previously described^30^. For total protein extracts, pea flour or frozen pea samples were ground with a mortar and pestle in 10 volumes of 50 mM Tris-HCl pH 8.0, followed by centrifugation at 16,000 × *g* for 10 min to remove debris. Samples were kept at 4 °C throughout the protein extraction procedure to minimize degradation. The protein concentration was determined using BioRad Protein Assay Dye Reagent and bovine serum albumin as a standard. Purified ferritin (5–40 ng) or pea extract (20 µg protein) were mixed with loading buffer (20 mM Tris-HCl pH 8.0, 80% (v/v) glycerol, 0.1% (w/v) bromophenol blue) and separated on a non-denaturing gel of 6% (w/v) acrylamide:bis-acrylamide (37.5:1) in 0.375 M Tris-HCl pH 8.0 for 2 h at 30 mA. Duplicate gels were either stained for protein with Instant Blue (Expedeon) or for iron with enhanced Perls’/diaminobenzidine (DAB) staining^31^. For the latter, gels were incubated in 0.75% (w/v) HCl and 2% (w/v) ferrocyanide in H_2_O for 20 min, then rinsed 4 × 5 min in MilliQ H_2_O. Next, gels were incubated in 0.075% (w/v) DAB, 0.015% H_2_O_2_ for 20 min. Bands with a brown colour specific for Fe associated with ferritin started to appear within 10 min. When the colour was sufficiently developed, usually after 30 min, gels were rinsed in water (3 × 5 min). Gels were placed in ~10 ml water and kept at 4 °C for 2–3 days to further enhance the colour before digital imaging.

### Immuno-detection of ferritin

Polyclonal antibodies against purified pea ferritin were raised in rabbit (Covalab, Cambridge, UK). The antisera were tested on known amounts of purified pea ferritin and on total proteins extracted from mature peas with 10% (w/v) trichloric acid in acetone. For most other purposes, proteins were extracted with 50 mM Tris-HCl pH 8.0. Protein extracts were mixed with loading buffer (0.125 M Tris-HCl pH 6.8, 2% (w/v) sodium dodecyl sulfate (SDS), 10% (v/v) glycerol, 0.1% (w/v) bromophenol blue, 5% (v/v) 2-mercaptoethanol) and separated on a 12.5% denaturing SDS-polyacrylamide gel. Proteins were transferred onto nitrocellulose membrane using semi-dry blotting. Membranes were blocked in Tris-buffered saline (TBS), 1% (v/v) Tween-20 and 5% (w/v) skimmed milk (TBS-TM) for 1 h. Antibodies were diluted 1:5000 in TBS-TM and incubated for 1 h. Membranes were washed (3 × 5 min) with TBS-T, and then incubated with anti-rabbit IgG conjugated to horseradish peroxidase (Abcam UK, ab6721) at a 1:5000 dilution in TBS-T. After 4 × 5 min TBS-T washes, antibody binding was visualized with chemiluminescence reagents and digitally imaged using a LAS-500 fluorescent imager.

Immunolabelling of sections was as described^32^. The rabbit polyclonal antibodies against ferritin were detected using Alexa Fluor 488 goat anti-rabbit IgG (Invitrogen, A11008). Images were acquired with a Zeiss Axiophot epifluorescence microscope using a Retiga EXT CCD digital camera (QImaging, Canada) and Metamorph software (Molecular Devices, USA).

### Embedding and sectioning

Tissue samples of approximately 2 × 2 mm were infiltrated with 0.5 M MES-KOH pH 5.4, and high pressure frozen in 6 mm sample carriers using a high-pressure freezer (HPM 100 from Leica Microsystems, UK). Frozen samples were transferred to tubes containing frozen 100% (v/v) ethanol in liquid nitrogen and placed in an automatic freeze substitution system (EM AFS from Leica Microsystems, UK). Samples were sequentially warmed over 5 days to −30 °C, then to 4 °C over 48 h and finally to room temperature. The samples were processed through increasing concentrations of LR White resin (Agar Scientific UK, R1281) and embedded at 58 °C for 16–20 h in a nitrogen-rich environment. Sections (1 µm) of the resin blocks were cut with a Reichert-Jung ultramicrotome, and dried at 40 °C onto platinum-coated Thermanox coverslips for NanoSIMS, or on Polysine-coated slides (Agar Scientific UK, L4345) for immunolabelling.

### NanoSIMS

NanoSIMS analysis was performed with a Cameca NanoSIMS 50 L (Cameca, France). A 16 keV Cs^+^ ion beam with a current of 1.5–2.5 pA and a beam size of approximately 150 nm (D1 = 2, 300 µm aperture) was focused onto the sample and rastered over the surface to generate negative secondary ions. The ions sputtered from the sample were analysed in a mass spectrometer to generate ion images of the tissue. Ion maps were simultaneously collected for ^16^O^−^, ^12^C^14^N^−^, ^32^S^−^, ^31^P^12^C^−^, ^31^P^16^O^−^ and ^56^Fe^16^O^−^ (all aligned on high concentration standards) as well as the secondary electron map. For each area a dose of 1 × 10^17^ Cs^+^ ions cm^2^ was implanted before imaging by continuously scanning a large defocused beam to remove the platinum coating and maximize signal intensity. A 50 × 50 µm area was imaged with a dwell time of 5 ms per pixel. Sequential images from each region of interest were acquired and summed to improve the counting statistics. The number of images acquired was varied to give the same total dose for each region depending on the beam current. Image processing was conducted with ImageJ using the OpenMIMS plugin (Harvard).

### *In vitro* iron bioavailability studies

Garden peas were microwaved following the cooking instruction on the pack. In brief, 100 g frozen garden peas were placed in a beaker with 1 tablespoon (9.5 ml) MilliQ water (18.2 MΩ), and microwaved at 900 W for 2.5 min. Dried peas (100 g) were soaked in 500 ml Milli-Q water overnight, rinsed 3 times and boiled with 400 ml MilliQ water for 1 h. After cooking, samples were frozen at −80 °C, lyophilized, finely ground and stored at 4 °C until further use.

The simulated gastrointestinal digestion was performed as described^19^ with minor modifications. Pea samples were added to 10 ml of saline solution (140 mM NaCl, 5 mM KCl) at pH 2.0, followed by addition of pepsin (0.04 g ml^−1^) and incubated for 90 min on a rolling platform at 37 °C to simulate gastric conditions. Ascorbic acid was added at a molar ratio of 10:1 ascorbate:iron. Subsequently, the pH of the samples was gradually adjusted to pH 5.5 with NaHCO_3_. Bile extract (0.007 g ml^−1^) and pancreatin (0.001 g ml^−1^) were added, samples were readjusted to pH 7, and incubated for an additional hour on a rolling platform at 37 °C to mimic intestinal conditions. At the end of the simulated gastrointestinal digestion, samples were centrifuged at 3000 × *g* for 10 min and the supernatants were used for subsequent cell culture experiments. A volume of 1.5 ml supernatant was applied to an upper chamber consisting of a Transwell insert fitted with a 15 kDa molecular weight cut-off dialysis membrane (Spectra/Por 7 dialysis tubing, Spectrum Laboratories, Europe) suspended over Caco-2 cell monolayers grown in collagen-coated 6-well plates. After incubation for one hour at 37 °C in a humidified incubator containing 5% CO_2_ and 95% air, inserts were removed and an additional 1 ml of low-iron Eagle’s minimal essential medium (MEM, GIBCO, Grand Island, NY) was added. Cells were incubated for a further 23 hours prior to harvesting for ferritin analysis. Details of Caco-2 cell culture and ferritin analysis by ELISA are described in ref.^33^. To assess the effect of phytic acid on iron bioavailability, either 0.5 mM phytic acid was added to the digestate of microwaved garden peas, or 480 U phytase enzyme (Megazyme) was added to the digestate of boiled mature peas.

### Statistical analysis

Quantitative data are presented as mean values with standard errors of the means (SEM). Homogeneity of variance was tested using the Levene’s test. For multiple comparison, one-way ANOVA was used followed by a post-hoc Tamhane test for non-homogenous variances. The Student’s *t*-test was used when two treatments were compared to each other. Statistical significance was set at *p* < 0.05. The statistical analysis was performed using the SPSS package (version 23; SPSS Inc., Chicago, IL, USA).

### Data availability statement

All data generated or analyzed during this study are included in this published article or in the supplementary information.

## Electronic supplementary material

Supplementary Information
