# Supplementary material for: The stage of seed development influences iron bioavailability in pea (Pisum sativum L.)
Source: Sci Rep. 2018 May 2;8:6865. doi: 10.1038/s41598-018-25130-3 (PMC5932076; doi:10.1038/s41598-018-25130-3)
Supplement: Supplementary file 1 — Supplementary Information [file 41598_2018_25130_MOESM1_ESM.pptx]

## Slide 1
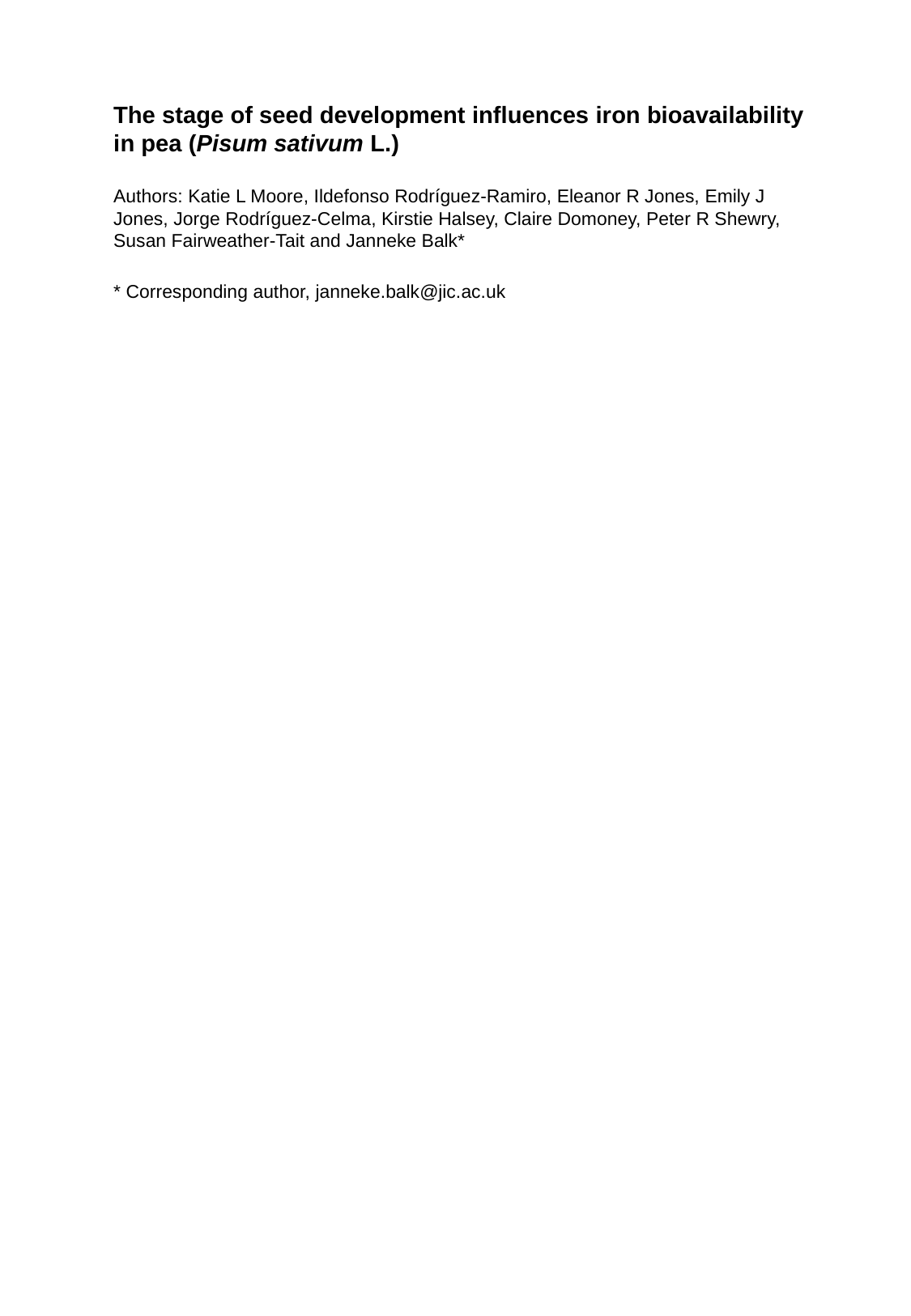

The stage of seed development influences iron bioavailability in pea (Pisum sativum L.)
Authors: Katie L Moore, Ildefonso Rodríguez-Ramiro, Eleanor R Jones, Emily J Jones, Jorge Rodríguez-Celma, Kirstie Halsey, Claire Domoney, Peter R Shewry, Susan Fairweather-Tait and Janneke Balk*
* Corresponding author, janneke.balk@jic.ac.uk

## Slide 2
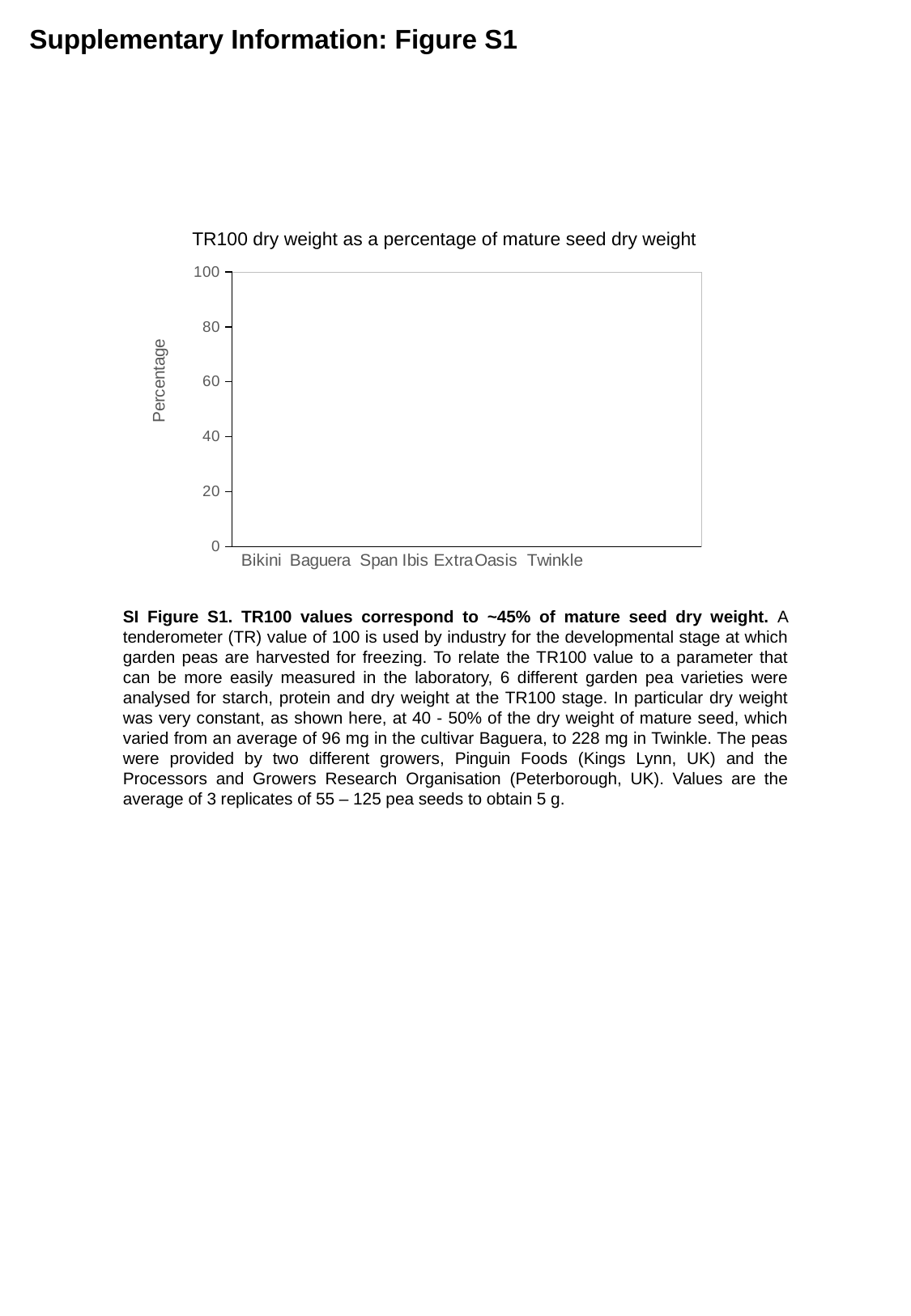

Supplementary Information: Figure S1
TR100 dry weight as a percentage of mature seed dry weight
### Chart
| Category | |
|---|---|
| Bikini | 42.11240422506909 |
| Baguera | 51.027778259328606 |
| Span | 51.35498347155294 |
| Ibis Extra | 41.393007276847506 |
| Oasis | 47.07674344771119 |
| Twinkle | 45.2129598408297 |SI Figure S1. TR100 values correspond to ~45% of mature seed dry weight. A tenderometer (TR) value of 100 is used by industry for the developmental stage at which garden peas are harvested for freezing. To relate the TR100 value to a parameter that can be more easily measured in the laboratory, 6 different garden pea varieties were analysed for starch, protein and dry weight at the TR100 stage. In particular dry weight was very constant, as shown here, at 40 - 50% of the dry weight of mature seed, which varied from an average of 96 mg in the cultivar Baguera, to 228 mg in Twinkle. The peas were provided by two different growers, Pinguin Foods (Kings Lynn, UK) and the Processors and Growers Research Organisation (Peterborough, UK). Values are the average of 3 replicates of 55 – 125 pea seeds to obtain 5 g.

## Slide 3
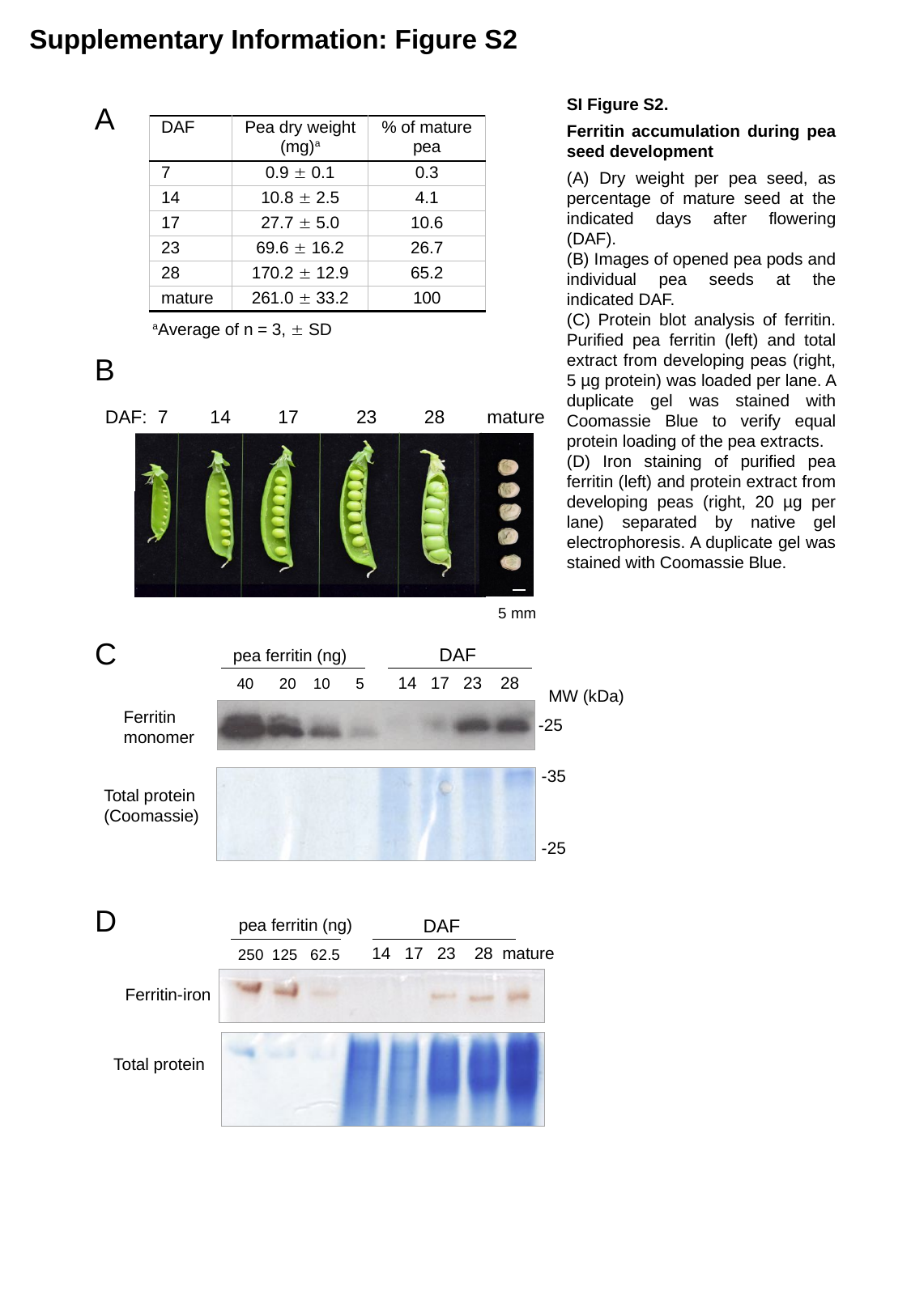

Supplementary Information: Figure S2
SI Figure S2.
Ferritin accumulation during pea seed development
(A) Dry weight per pea seed, as percentage of mature seed at the indicated days after flowering (DAF).
(B) Images of opened pea pods and individual pea seeds at the indicated DAF.
(C) Protein blot analysis of ferritin. Purified pea ferritin (left) and total extract from developing peas (right, 5 µg protein) was loaded per lane. A duplicate gel was stained with Coomassie Blue to verify equal protein loading of the pea extracts.
(D) Iron staining of purified pea ferritin (left) and protein extract from developing peas (right, 20 µg per lane) separated by native gel electrophoresis. A duplicate gel was stained with Coomassie Blue.
A
| DAF | Pea dry weight (mg)a | % of mature pea |
| --- | --- | --- |
| 7 | 0.9 ± 0.1 | 0.3 |
| 14 | 10.8 ± 2.5 | 4.1 |
| 17 | 27.7 ± 5.0 | 10.6 |
| 23 | 69.6 ± 16.2 | 26.7 |
| 28 | 170.2 ± 12.9 | 65.2 |
| mature | 261.0 ± 33.2 | 100 |
aAverage of n = 3, ± SD
B
DAF: 7 14 17 23 28 mature
5 mm
C
DAF
pea ferritin (ng)
14 17 23 28
40 20 10 5
MW (kDa)
Ferritin monomer
-25
-35
Total protein (Coomassie)
-25
D
DAF
pea ferritin (ng)
14 17 23 28 mature
250 125 62.5
Ferritin-iron
Total protein

## Slide 4
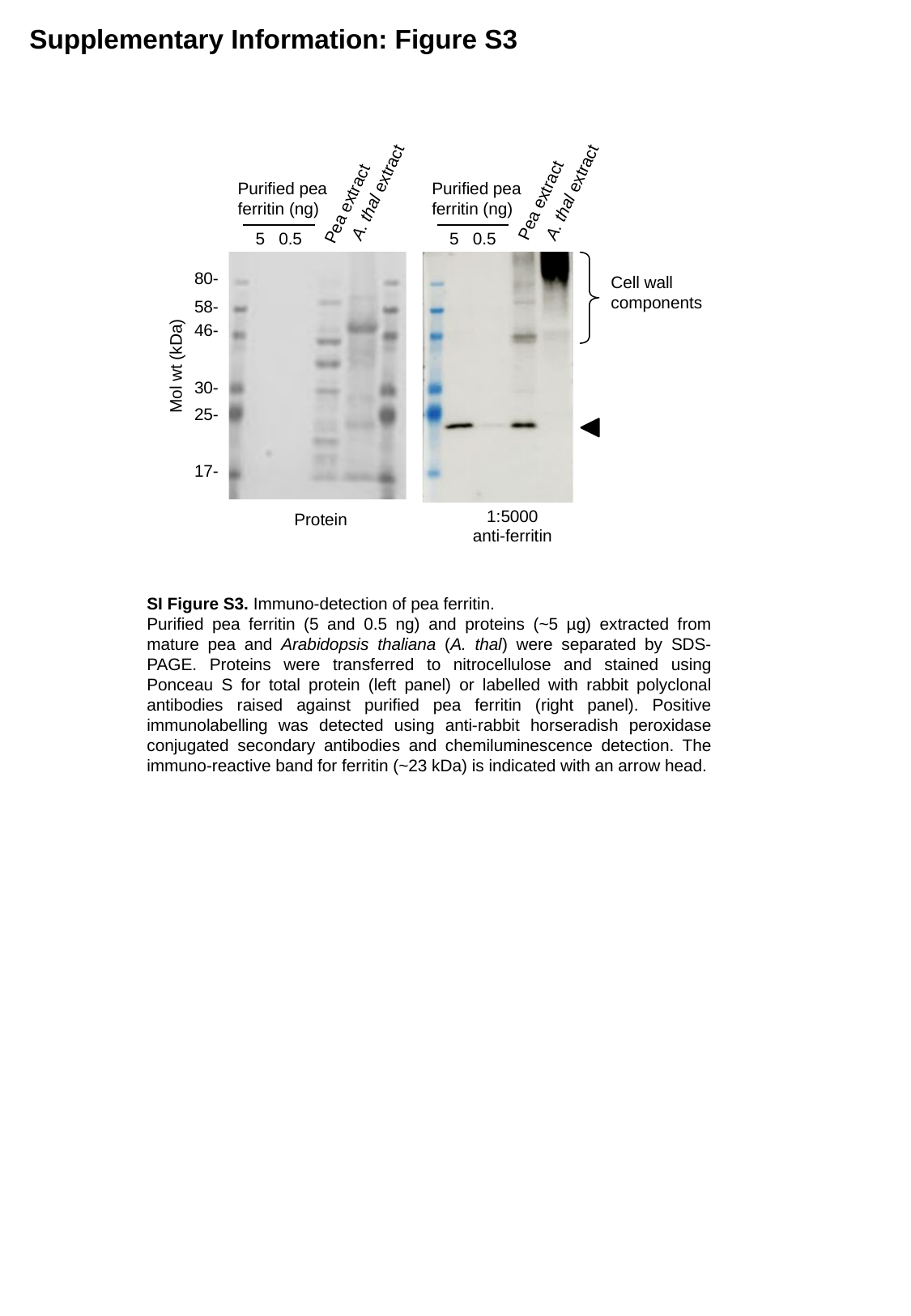

Supplementary Information: Figure S3
Purified pea ferritin (ng)
Pea extract
5 0.5
A. thal extract
A. thal extract
Purified pea ferritin (ng)
Pea extract
5 0.5
80-
Cell wall components
58-
46-
Mol wt (kDa)
30-
25-
17-
1:5000
anti-ferritin
Protein
SI Figure S3. Immuno-detection of pea ferritin.
Purified pea ferritin (5 and 0.5 ng) and proteins (~5 µg) extracted from mature pea and Arabidopsis thaliana (A. thal) were separated by SDS-PAGE. Proteins were transferred to nitrocellulose and stained using Ponceau S for total protein (left panel) or labelled with rabbit polyclonal antibodies raised against purified pea ferritin (right panel). Positive immunolabelling was detected using anti-rabbit horseradish peroxidase conjugated secondary antibodies and chemiluminescence detection. The immuno-reactive band for ferritin (~23 kDa) is indicated with an arrow head.

## Slide 5
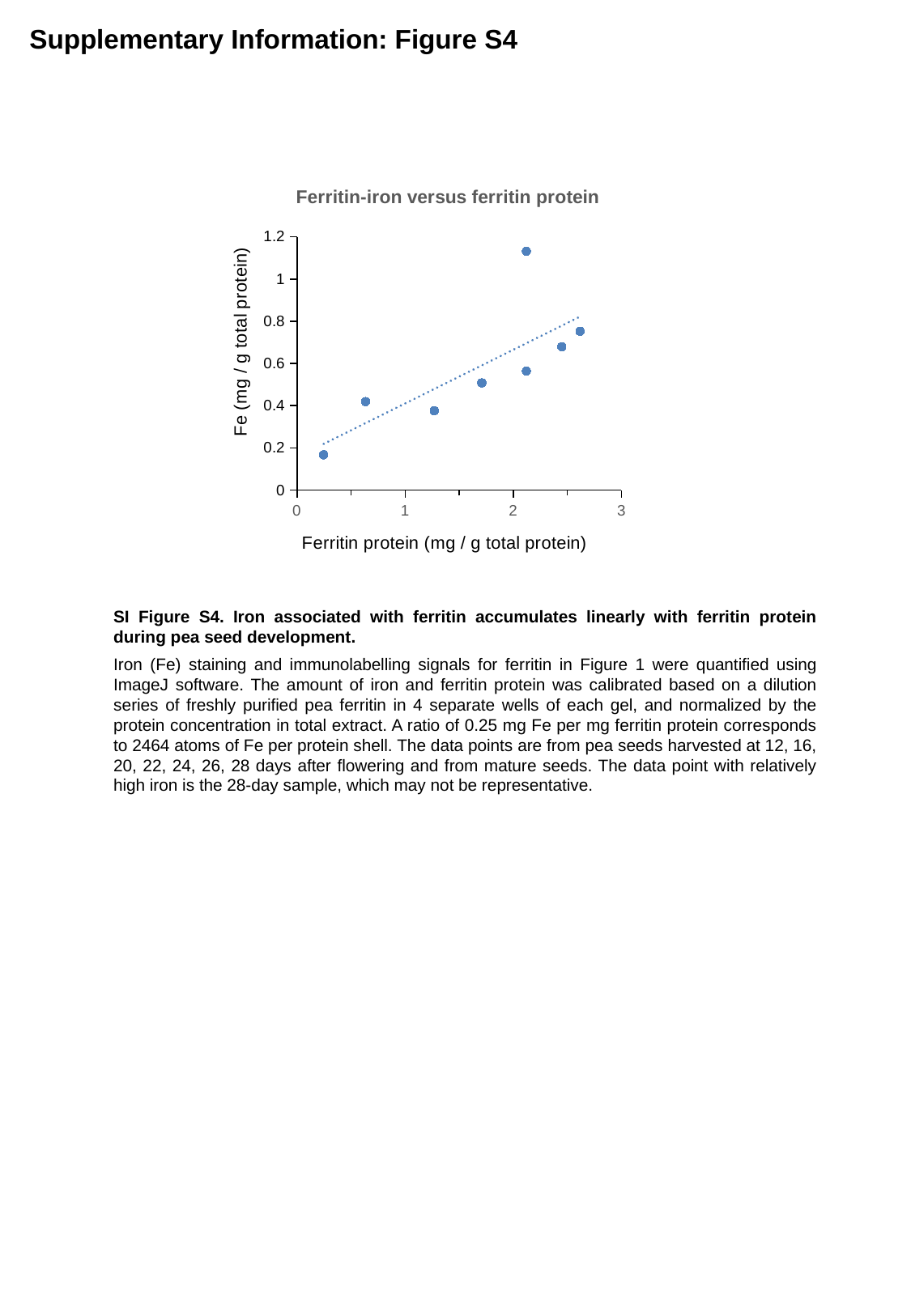

Supplementary Information: Figure S4
### Chart: Ferritin-iron versus ferritin protein
| Category | |
|---|---|SI Figure S4. Iron associated with ferritin accumulates linearly with ferritin protein during pea seed development.
Iron (Fe) staining and immunolabelling signals for ferritin in Figure 1 were quantified using ImageJ software. The amount of iron and ferritin protein was calibrated based on a dilution series of freshly purified pea ferritin in 4 separate wells of each gel, and normalized by the protein concentration in total extract. A ratio of 0.25 mg Fe per mg ferritin protein corresponds to 2464 atoms of Fe per protein shell. The data points are from pea seeds harvested at 12, 16, 20, 22, 24, 26, 28 days after flowering and from mature seeds. The data point with relatively high iron is the 28-day sample, which may not be representative.

## Slide 6
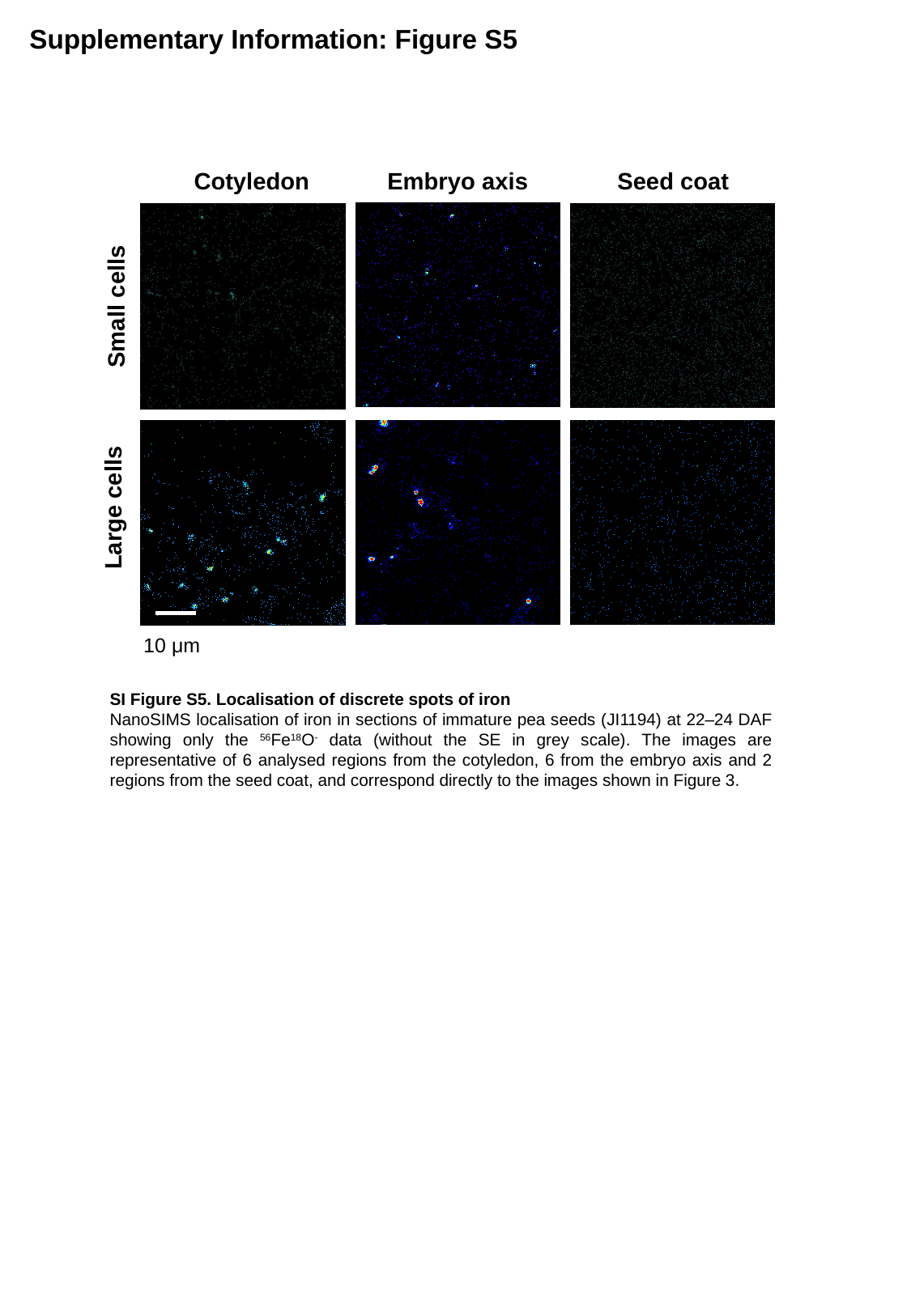

Supplementary Information: Figure S5
Embryo axis
Seed coat
Cotyledon
Small cells
Large cells
10 μm
SI Figure S5. Localisation of discrete spots of iron
NanoSIMS localisation of iron in sections of immature pea seeds (JI1194) at 22–24 DAF showing only the 56Fe18O- data (without the SE in grey scale). The images are representative of 6 analysed regions from the cotyledon, 6 from the embryo axis and 2 regions from the seed coat, and correspond directly to the images shown in Figure 3.

## Slide 7
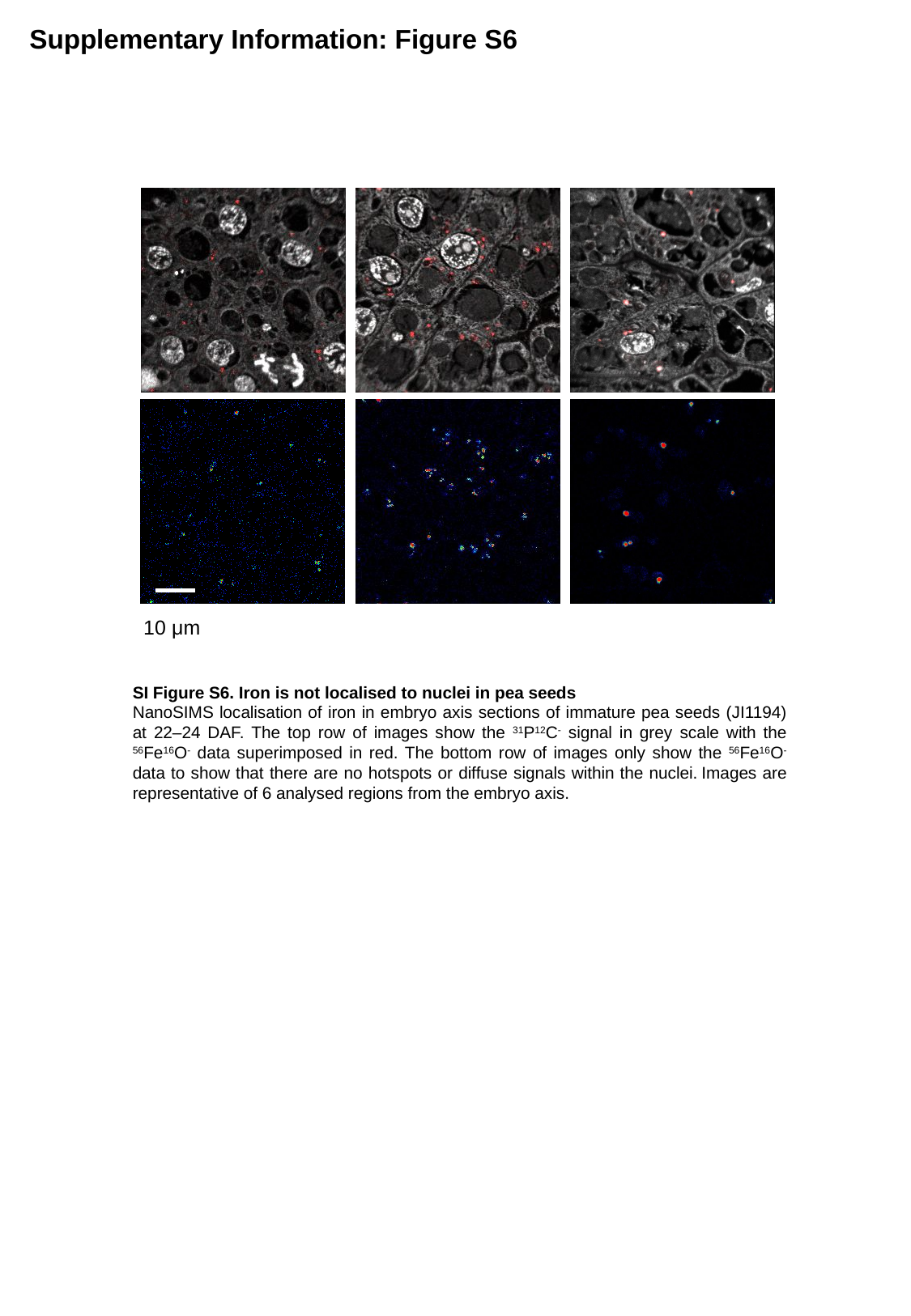

Supplementary Information: Figure S6
10 μm
SI Figure S6. Iron is not localised to nuclei in pea seeds
NanoSIMS localisation of iron in embryo axis sections of immature pea seeds (JI1194) at 22–24 DAF. The top row of images show the 31P12C- signal in grey scale with the 56Fe16O- data superimposed in red. The bottom row of images only show the 56Fe16O- data to show that there are no hotspots or diffuse signals within the nuclei. Images are representative of 6 analysed regions from the embryo axis.
